# Consumers attitudes and beliefs towards the receipt of antenatal corticosteroids and use of clinical practice guidelines

**DOI:** 10.1186/s12884-016-1043-4

**Published:** 2016-09-05

**Authors:** E. L. McGoldrick, T. Crawford, J. A. Brown, K. M. Groom, C. A. Crowther

**Affiliations:** 1 Liggins Institute, The University of Auckland, Auckland, New Zealand; 2 Department of Obstetrics and Gynaecology, The University of Auckland, Auckland, New Zealand; 3 National Womens Health, Auckland City Hospital, Auckland, New Zealand

**Keywords:** Consumer, Implementation, Clinical practice guidelines, Antenatal corticosteroids, Theoretical domains framework

## Abstract

**Background:**

Active participation of consumers in health care decision making, policy and clinical research is increasingly encouraged by governments, influential bodies and funders. Identifying the best way to achieve this is difficult due to the paucity of evidence. Consumers have mixed feelings towards clinical practice guidelines (CPG) demonstrating scepticism towards their purpose and applicability to their needs. There is no information pertaining to consumers’ views and attitudes on the receipt of antenatal corticosteroids (ACS). The aim of this study was to examine the barriers and enablers to receiving ACS and use of CPG amongst consumers.

**Methods:**

Consumers were recruited from neonatal units across three district health boards (DHBs) in Auckland, New Zealand. Participants completed a semi-structured interview or questionnaire. The questions posed and analyses were informed by the Theoretical Domains Framework (TDF). Barriers and enablers were identified by the presence of conflicting beliefs within a domain; the frequency of beliefs; and the likely strength of the impact of a belief on use of CPG and receipt of ACS.

**Results:**

Twenty four consumers participated in the study. Six domains were identified as barriers to receipt of ACS and use of CPG. Key barriers to receipt of ACS included: difficulty retaining information conveyed, requiring further information in a variety of formats, and time constraints faced by consumers and health professionals in the provision and understanding of information to facilitate decision making. Barriers to use of CPG included: uncertainty about applicability of guideline use among consumers and scepticism about health professionals adhering too rigidly to guidelines. Enablers to receipt of ACS included: optimism toward ACS use, a strong knowledge of why ACS were administered, improved resilience in their pregnancy and confidence in their decision making following receipt of information about ACS. Enablers to use of CPG included: validation and standardisation of decision making among health professionals providing care and facilitating the best care for women and their babies.

**Conclusions:**

Key barriers and enablers exist among consumers regarding receipt of ACS and use of CPG. These need to be addressed or modified in any intervention strategy to facilitate implementation of the ACS CPG.

**Electronic supplementary material:**

The online version of this article (doi:10.1186/s12884-016-1043-4) contains supplementary material, which is available to authorized users.

## Background

Preterm birth remains a leading cause of perinatal morbidity and mortality worldwide [1]. Despite significant efforts the overall rates of preterm birth remain unchanged or are increasing [2]. Therefore optimising the outcomes of preterm infants’ remains essential [1].

A number of key interventions, including the administration of ACS have been identified to help mitigate the short and long-term effects associated with preterm birth [1]. A substantial body of evidence has demonstrated that a single course of ACS administered to women within 7 days prior to preterm birth can significantly reduce the risk of morbidity and mortality in their preterm infants. This includes a reduction in neonatal death, respiratory distress syndrome, necrotising enterocolitis, intraventricular haemorrhage and systemic infection within the first 48 hours of life [3]. Repeat doses(s) of ACS can be administered to women identified at on-going risk of preterm birth 7 days or more after administration of an initial course, to significantly reduce respiratory distress syndrome and a composite of serious infant outcomes [4]. A new ACS guideline entitled: “Antenatal corticosteroids given to women prior to birth to improve fetal, child and adult health”[5] used gold standard methods for guideline development [6, 7] to summarise evidence and provide recommendations around key clinical questions related to ACS administration [5].

Despite a large number of surveys being conducted assessing health professionals practices related to ACS [8–12] to the best of our knowledge there is no research available on consumer views related to the use of ACS for improving outcomes after preterm birth. Consumers have been identified as one of the key stakeholders in implementation of the new ACS CPG [5]. A ‘consumer’ has been defined as: “someone who uses, is affected by, or who is entitled to use a health related service; a relative or carer; or someone who advocates on behalf of broader group of people” [13]. Involving consumers in health care research has the potential benefits of prioritising research questions and identifying relevant clinical outcomes from the perspective of the consumer and their families [14]. Consumer participation in healthcare policy can facilitate public accountability and transparency [15, 16]. Consumers can also be actively involved in their own clinical care to facilitate shared decision making and empowering them to make more informed choices [17–19].

Governments and influential bodies such as the World Health Organisation, the Cochrane Collaboration [20], the National Institute for Health and Care Excellence (NICE) [21], the Consumers’ Health Forum of Australia [22] and the Guidelines International Network [23] have increasingly recognised the importance of consumer involvement in health care [14]. The World Health Organisation’s declaration of Alma Ata states that “People have the right and duty to participate individually and collectively in the planning and implementation of healthcare” [24].

In spite of organisations advocating for consumers to be actively involved in all levels of healthcare service, a recent Cochrane systematic review was unable to draw any definite conclusions on how best to achieve effective consumer involvement in the development of healthcare policy and research, CPG and patient information due to the paucity of evidence [16]. The authors identified the need for further research to address this [16].

A systematic review assessing the role of patient and public involvement in developing and implementing CPGs identified that patients believed their greatest impact would be in defining key questions to be addressed by CPGs, helping to formulate recommendations and in revising drafts of the guidelines to incorporate patients’ values or perspectives [25]. This mixed model systematic review included 26 eligible studies (10 qualitative studies, 13 cross sectional studies and three randomised controlled trials) and also identified that consumers demonstrated mixed feelings towards guidelines, with some scepticism as to their purpose and their applicability to their particular needs. A significant number of the participants were unaware of the existence of guidelines [25].

There has been increasing research to improve applicability and readability of CPG to facilitate dissemination, implementation and use of guidelines amongst consumers [26]. A number of organisations and professional groups have produced consumer versions of CPG recommendations to facilitate the provision and communication of information [21, 27, 28]. The Guidelines International Network has been particularly active in improving consumer involvement by producing a methodological tool kit to provide practical advice to guideline developers using the published literature and personal experience [29].

Unfortunately the uptake and adoption of evidence into clinical practice can be somewhat slow and haphazard [30]. CPGs can only improve or facilitate appropriate administration of ACS if the recommendations are adopted into routine clinical practice. The current literature suggests that an active tailored implementation strategy to address identified barriers is more likely to improve professional practice than dissemination of the guidelines alone [31].

Barriers and enablers are determinants of healthcare practice that might prevent or facilitate improvements in practice by modifying or mediating behaviours [32]. Barriers can exist at a political, organisational, individual and patient level [33]. A gap in the current literature pertaining to the assessment of barriers and enablers to implementation is that researchers focus on individual health professional barriers and often neglect the healthcare team, the organisation and the patient [34, 35].

The use of a theoretical framework can help to identify and understand the behavioural barriers and enablers that need to be altered or enhanced to facilitate effective implementation [36]. Modelling interventions to address pre-specified barriers or enhance enablers should facilitate understanding how and why interventions are successful [36]. A significant difficulty faced by researchers is to identify which theory to use [37]. The TDF has been developed to integrate 128 constructs from 33 health and social psychology theories that may explain health related behaviour change [36]. This framework has recently been validated and includes 14 behavioural domains and their component constructs [38] (Table 1). The TDF has previously been used in health care to understand consumers’ experiences in taking and adhering to medication and lifestyle modifications to prevent or optimise disease management [39–44].

**Table 1:** Theoretical domains framework, adapted from Cane et al. [36, 38]

Theoretical domains	Definition
Knowledge	An awareness of the existence of something
Skills	An ability or proficiency acquired through practice
Social/Professional Role & Identity	A coherent set of behaviours and displayed personal qualities of an individual in a social or work setting
Beliefs about capabilities	Acceptance of the truth, reality or validity about an ability, talent or facility that a person can put to constructive use
Optimism	The confidence that things will happen for the best or that desired goals will be attained
Beliefs about consequences	Acceptance of the truth, reality or validity about outcomes of a behaviour in a given situation
Reinforcement	Increasing the probability of a response by arranging a dependent relationship, or contingency, between the response and a given stimulus
Intentions	A conscious decision to perform a behavior or a resolve to act in a certain way
Goals	Mental representations of outcomes or end states that an individual wants to achieve
Memory, attention and decision processes	The ability to retain information, focus selectively on aspects of the environment and choose between two or more alternatives
Environmental context and resources	Any circumstance of a person’s situation or environment that discourages or encourages the development of skills and abilities, independence, social competence, and adaptive behaviour
Social influences	Those interpersonal processes that can cause individuals to change their thoughts, feelings or behaviours
Emotion	A complex reaction pattern, involving experiential, behavioural and physiological elements, by which the individual attempts to deal with a personally significant matter or event
Behavioural Regulation	Anything aimed at managing or changing objectively observed or measured actions

The aim of this study was to identify consumers’ attitudes, beliefs and knowledge on the receipt of ACS and use of CPGs and to determine their views on barriers and enablers to their use, to inform implementation strategies for the ‘Antenatal corticosteroids given to women prior to birth to improve fetal, child and adult health’ clinical practice guideline (CPG guideline 2015) [5].

## Methods

Design: This was a mixed method study using in-depth, semi-structured interviews and on-line questionnaires.

### Setting

We recruited participants from four maternity hospitals in Auckland, New Zealand. These hospitals reflect different levels of maternal and newborn care and capture the social and ethnic diversity of three distinctly different DHBs in Auckland, New Zealand.

These three DHBs serve a catchment population of approximately 1,575,380 individuals (34 % of New Zealand population) based on data projections from the 2013 census [45]. Collectively the populations served by the three DHBs are reflective of the overall population of New Zealand [46].

National Women’s Health (Auckland DHB) and Middlemore Hospital (Counties Manukau DHB) provide care to babies who need full ventilation, or are born at less than 30 weeks’ gestation or who require intensive care support. The neonatal service at National Women’s Health receives babies from other New Zealand regions and cares for babies requiring neonatal surgery and other specialist services. North Shore and Waitakere Hospitals (Waitemata DHB) care for women with low- or medium-risk pregnancies and their special care baby units can accommodate babies from 32 weeks’ gestation onwards.

### Participants

Participants were recruited postnatally from the three DHBs within Auckland, New Zealand. Women were eligible to participate if they had received a dose(s) of ACS prior to preterm birth at or less than 34 weeks’ gestation.

Potential participants were identified by a member of the neonatal or obstetric team at each of the recruiting hospital sites and invited to take part by one of the researchers (EM). A participant information sheet was provided and written consent was obtained from all participants prior to taking part in the study. Participants were randomised into completing either a semi-structured interview or questionnaire. This study was nested within a randomised trial to assess different methods for identifying barriers and enablers to administration of antenatal corticosteroids. The results from this comparison will be reported elsewhere.

Eight individuals were recruited at each of the three DHBs to facilitate data saturation in the thematic analysis. Ethical approval was obtained by the University of Auckland Human Participants Ethics Committee (011193) and locality agreement was obtained at each of the participating sites.

### Materials

The TDF [36, 38] informed the development of the questions used in both the semi-structured interviews and on-line questionnaires (Additional file 1). The questions were developed to provide uniformity of assessment in eligible groups (semi-structured interview and questionnaire) and were piloted by three consumers to ensure clarity of the questions and minimise repetition.

The questions explored:

1. Attitudes, beliefs and knowledge on the receipt of ACS
2. Knowledge and beliefs of CPGs;
3. Additional resources required to facilitate decision making

### Interview procedure

Semi-structured, face to face interviews were conducted by a single researcher (EM) who had training in interview skills. Interviews were conducted in the neonatal unit at the respective hospital sites, at a time convenient to the women and their partners. No explicit time restraints were applied for the interviews.

### Questionnaire procedure

The questionnaires were developed on the web based system, Survey Monkey®, and were emailed to the participants preferred email address to be completed at a time convenient to them. For participants without access to a computer a paper version of the questionnaire was provided and collected following completion. Participants were asked to insert their unique identifying number at the beginning of the questionnaire to allow identification of responders. A reminder via email or text message was sent to non-responders 2 weeks after the initial questionnaire had been sent.

### Data collection and analysis

The semi-structured interviews and online questionnaires were conducted over an eight month time period (April to November 2014). Following each interview, the digital recordings were transcribed verbatim by a single researcher (EM). The transcripts were verified by another researcher (TC) and entered into NVivo8 [47] for data management and analysis. The transcripts were read and re-read by both reviewers (EM, TC) to familiarise themselves with the data. A deductive process of thematic analysis was used to classify responses into one or more of the 14 theoretical domains using the TDF [36, 38]. Both reviewers coded the semi-structured interview data and open ended questionnaire responses independently. This process was iterative and involved discussion between the two reviewers and consultation with the other members of the research team (CAC, KG, JB). Lists of specific beliefs were then generated to represent the overarching statements coded within each behavioural domain [48]. The two reviewers decided whether the beliefs would represent a barrier or enabler to receipt of ACS and use of CPG. A frequency count illustrated the strength of beliefs within domains.

Data saturation was assessed throughout sampling, data collection and data analysis. No further participants were required after initial recruitment as no new themes were emerging from the data.

## Results

**Fig. 1:**
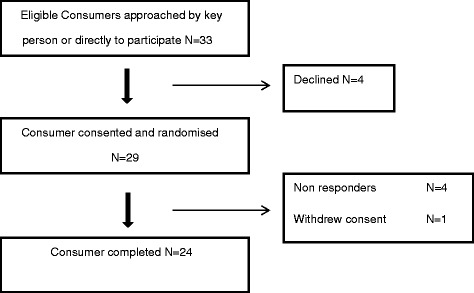
Flowchart of recruitment

A total of 24 consumers completed either a semi-structured interview (11) or questionnaire (13) (Fig. 1). Participants were predominantly aged between 30 and 39 years (58 %), of New Zealand European ethnicity (46 %) and had received a single complete course of ACS (54 %) (Table 2). All participants had given birth prior to 34 weeks’ gestation and the commonest co-morbidity reported was preterm prelabour rupture of membranes (PPROM) (18 %). Of those women completing the interview 7/11 (64 %) were interviewed alone, the remainder had their partner or another family member in attendance. Our study population is representative of the cohort of mothers and babies admitted to neonatal units across New Zealand in relation to maternal age (ANZNN 2013: 49 % of level 3 registrants aged 30–39 years) ethnic diversity (ANZNN 2013: 52.3 % level 2 and 52.6 % of level 3 registrants were Caucasian), and indications or reasons for preterm birth (ANZNN 2013: PPROM 18 % level 2 and 3 registrants) when compared to data from the Australia and New Zealand Neonatal Network data [49].

**Table 2:** Demographics of participants included in the study

Age group (years)	Number (% of total)
< 20 years	0
20–29	3 (13 %)
30–39	18 (75 %)
≥ 40	3 (13 %)
Ethnicity	
European	11 (46 %)
Maori	5 (21 %)
Pacific Island	4 (17 %)
Asian	3 (13 %)
Middle Eastern/Latin American/ African	1 (4 %)
Employed	
Yes	18 (75 %)
Co-morbidities in pregnancy (by consumers)	
Premature rupture of membranes	11 (46 %)
Multiple pregnancy	10 (42 %)
High blood pressure/pre-eclampsia	4 (17 %)
Antepartum Haemorrhage	4 (17 %)
Diabetes	3 (13 %)
Intrauterine growth restriction	2 (8 %)
Other^a^	4 (17 %)
Complete course of antenatal corticosteroids	
Yes	18 (75 %)
No	2 (83 %)
Unsure	4 (17 %)

^a^Other: 2 participants’ twin-twin transfusion and 2 participants had a cervical cerclage

### Barriers and enablers

The behavioural domain of environmental context and resources was identified as a barrier to the receipt of ACS. Some behavioural domains were identified as barriers and enablers to the receipt of ACS. The four behavioural domains identified as mixed barriers and enablers to the receipt of ACS included: knowledge, memory attention and decision processes, emotion and belief about capabilities. Five additional behavioural domains were identified as enablers alone, to receipt of antenatal corticosteroids. These were: optimism, social influences, goals, belief about consequences and behavioural regulation.

One behavioural domain (social professional role and identity) was identified as a barrier and enabler to the use of CPG. A further three behavioural domains were identified as enablers alone. These were: knowledge, optimism and goals.

The barriers and enablers to receipt of ACS and use of CPG are discussed in further detail within their respective behavioural domains. Key responses are demonstrated by direct quotes from participants. Theoretical domains not linked to barriers or enablers were skills, reinforcement and intentions.

### Environmental context and resources

This domain reflects the situation or circumstance that influences the individual’s ability to make decisions. Participants reported time constraints as a significant barrier to receiving and understanding the information on ACS. This was reflective of the acute nature of the situation in which they presented, necessitating urgency in both their and the health professionals decision making.

*“So I can’t remember a lot of thing they said because at the time it’s like,… you know painful and then at the same time the nurse comes and says…ah this medicine is good” (site 3, participant 3).*

However on occasion consumers had been identified as high risk of preterm birth early in their pregnancy and been seen by members of the health professional team. Consumers expressed the preference to receive the information as soon as possible to facilitate their decision making.

“So you could have a read through but obviously, something like when your waters break you don’t have enough time to go through that. But yeah, for those ladies they know their placenta is small or they are probably going to have little babies….something is going to happen. So in that case leaflets would be very good” (site 3, participant 4).

“I suppose it’s hard because I was preterm, it would be nice to get it earlier maybe through antenatal classes, as at the time you are under pressure” (site 2, participant 6).

This was linked to the belief that once consumers had been identified as high risk they had limited contact with their lead midwife and their care was primarily with the hospital health professional staff and therefore felt very reliant on a limited number of individuals for their information.

*“No my midwife after I got pregnant I think I saw her only about two times. After I give birth to the baby I think I only saw her last week. I didn’t see her much” (Site 1, participant 7).*

*“Probably the ones in hospital because I didn’t have much to do….I only had one appointment with my midwife. Yeah, the obstetrician, even though she was really good we didn’t get a chance to discuss much” (Site 2, participant 1)*

### Knowledge

Within our study, this domain reflected participant’s knowledge of the receipt of ACS including the name of the ACS administered, the dose and number of courses received and why they were administered. The majority of participants (21/24, 88 %) were aware that ACS were administered to improve fetal lung maturity.

*“Doctors and my obstetrician explained that due to my membranes breaking at 20 weeks’ the steroids would help with my babies lung development (site 2, participant 2)*

Many women identified that they were aware of the number of injections (courses) they received but were unsure of the name of the ACS used or the dose administered and would like this information in the future if ACS were required.

*“I was given info on all processes throughout treatment as well as during the procedure of my caesarean however I am unsure on the exact injections and/or medication used at present” (site 3, participant 8).*

The knowledge domain also referred to consumers’ awareness of the existence of CPG and why they were used. When asked about the use of CPG in assisting health professionals to make decisions about their care, a large proportion of participants were aware of the use of guidelines (20/24, 83 %) and why and how they should be used (17/24, 71 %).

*“Making clear about the time to give and the specific purpose,…clarifying how to use them” (site1, participant 1).*

### Memory attention and decision processes

This domain reflected the ability of the consumer to retain the information they received regarding ACS and use this to make decisions about their care. This domain appeared to represent the most significant barrier related to the understanding of the use of ACS. The majority of consumers (14/23, 61 %) expressed the belief that in a future pregnancy they would like more information regarding ACS and their administration. The degree of information required varied as reflected in the comments:

*"What could be good is exactly why the steroids are given, the names of the steroids say and the impact physiologically on the child and the mother and how that, how that hmm, how that would develop as your number of doses increased or something. So I don’t know. I know I had steroids but I don’t know exactly what. Whereas I normally would find out I think" (site 1, participant 2).*

*“Hmm, personally in this sort of situation because I know so little myself I have to rely on the professionals. Because I have already been through it, I would probably investigate a bit more” (site 3, participant 7).*

A number of participants (11/24, 46 %) remembered being given the information but due to the nature of the clinical situation they cannot necessarily recall everything that was said.

*“The information given at the time was all that was necessary and explained very well, although it is again hard to remember as it was an emergency situation and I was scared of the outcome” (site 3, participant 8).*

Consumers identified that having the information in a written format alongside verbal communication would facilitate understanding and retention of what was said.

*“Written material would be useful, antenatal classes: mention preterm labour and add in information about steroids” (site 2, participant 3) (in response to the question: In a future pregnancy would you find it helpful to receive more information)*

*“Something written would be good. Hmm just so you can…Hmm you know you don’t take it all in when someone is telling you something. But if you can sit there and read something, you know” (site 2, participant 1).*

### Emotion

This domain represents the reaction patterns with which the individual responds to a personally significant matter or event. This domain was represented strongly in the interviews and surveys completed by the participants. A significant barrier reported by participants to their usual decision making processes was the fear they experienced in being identified as at risk or being in preterm labour and the impact this had on their decisions. This is reflected by the comments:

*“I think it was because it was late at night and I was tired and freaked out” (site 2, participant 6).*

*“we were quite frantic” (site 1, participant 5).*

On occasion, frustration was expressed at the appreciated uncertainties in the evidence base and the impact this had on both them and their health professionals’ decision making.

*"And no one knows which one is right. You are thinking well that’s no help what so ever" (site 1, participant 2).*

Consumers expressed confidence in their decision making when they had been given adequate information on when and how ACS should be administered.

*“Because you can read that and you kind of know what you are in for” (site 2, participant number 2).*

*“So it might be a little bit familiar to you, you can think ahh, I have actually heard that before. So you are more confident in the decision that you are making” (site 2, participant 6)*

### Belief about capabilities

Within our study, this domain refers to an individuals’ ability to use their knowledge on ACS to make decisions about their care. A notable barrier within this domain was the belief by one individual that it was the doctors that made the decision on ACS administration rather than themselves.

“*Well I did not take that decision it was made by the doctors. They explained it to me but yeah I did not have the decision” (site 2, participant 8).*

However the majority of participants (23/24, 96 %) felt engaged in the decision making process and believed that they were able to use their own judgement to make the decision on whether to receive ACS with input from the clinical team.

“A the end of the day I make my decisions” (site 3, participant 5).

Another enabler identified within this behavioural domain was that consumers felt empowered when they received adequate information on ACS and reported that having the information on ACS allowed them to be confident and informed in the decision they were making.

*“So if you just gather as much information as you can then you are as informed as you can be” (site 2, participant 6).*

Interestingly some participants reported some difficulty in their decision making. This occurred when participants were informed of the uncertainties in the evidence base as perceived by their health professionals relating to the use of repeat dose(s)/course(s) of ACS.

*“It was a good conversation but it was like, there is no right or wrong on this. Which I assume there isn’t because everyone seems to have their own opinion” (site 1, participant 2).*

### Optimism

This domain reflected individuals’ confidence that administration of ACS happens for the best and that adherence to CPG will result in the desired goals being obtained. This domain represented a key enabler to ACS use. All participants considering a future pregnancy reported that they would be happy receiving further course(s) or dose(s) of ACS if they were at risk of a preterm birth in a subsequent pregnancy.

*“I was pretty determined that it was the right thing……..and they actually helped with some of my pregnancy symptoms, my swelling went down and I felt a lot better about myself” (site 1, participant 5).*

*Yes, I would…I’d like to get them earlier if I could” (site 3, participant 6).*

### Social Influences

This domain referred to the interpersonal processes that assisted consumers in making their decisions regarding receipt of ACS. Consumers reported that they relied very much on the medical staff when they were making decisions about their care and the care of their baby.

*“Usually the doctors or the nurses, they advise me what it is for. They are the most important ones because they know it better and I feel they wouldn’t give me the wrong advice” (site 3, participant 5).*Consumers identified that they also received support from family members and their partners when making important decisions about their care.

“Yeah my other sister, she has got two babies as well. She is the one that explains everything about having a baby. So she knows lots of things, so I ask her that,…..so what the next step is and she helps me” (site 3, participant 3).

### Goals

This domain reflects the mental representations of outcomes or end states that an individual wishes to achieve. Consumers were asked what they thought the purpose of the new ACS guidelines should be. Among individuals who were aware of CPG (20/24, 83 %), a consistent statement was that they felt adherence to CPG provided health professionals with a structure and ensured validation and standardisation of their decision making (7/17, 41 %).

*“Well I mean they need to have a structure…I guess validate them and not just make a big decision, without sort of checking them and validating them through a regulated higher party" (site 1, participant number 5).*

*“To have a general rule across the board of the administration of steroids” (site 2, participant 5).*

### Beliefs about consequences

This domain represents acceptance of truth, reality or validity about outcomes of a behaviour in a given situation. Within our study this relates to consumers views on the efficacy of ACS and their use in preterm birth. A significant proportion of the consumers (22/24, 92 %) felt that ACS were hugely beneficial in the treatment of preterm babies.

“I felt that they really helped my son” (site 3, participant 5).

Overall the majority of consumers (23/24, 96 %) were unable to identify any particular reasons they would not be happy receiving antenatal corticosteroids in a future pregnancy if indicated. However, two consumers were mindful of the need to ensure that ACS are only given when appropriate and emphasised that consideration needs to be taken in preventing unnecessary use and informing women of potential side effects or adverse reactions associated with antenatal corticosteroid administration.

“Maybe a reference to a website so that I could read more about the side effects etc” (site 2, participant 2).

“Hmm not if I knew that they were preterm,… or unless some research came out that very strongly suggested otherwise” (site 1, participant 2)

### Behavioural regulation

This domain refers to anything aimed at managing or changing objectively observed or measured actions. Specifically consumers were asked if in a future pregnancy they would like to receive any further information. As mentioned within the domain memory, attention and decision processes a significant proportion requested more information (14/23, 61 %). Additionally a number of participants also expressed that having time and information about ACS administration helped to facilitate their decision making.

*“I think it is important to know what they are doing and why. Ah…first my doctor in XX said it was an option to do it if the baby arrived before 34 weeks. Later here when the lady gave the steroids she explained before and I think we read some information the doctor gave me” (site 1, participant 4)“*

*“I thought, that we could of talked about it right at the start…worst case. But the thing is we didn’t know what was going to happen…..But I guess in hindsight maybe just mention it, There’s you know….there is good precautions like steroids that could help the twins” (site 1, participant 5).*

### Social professional role and identity

This domain reflects the individuals’ behaviours and perceived identity within their current situation or setting. However, when consumers were asked questions regarding their own social professional roles and identity many reflected what their expectations of their health professionals was, as opposed to their role in the use of CPG or in their decision making around ACS. When asked what they thought the purpose of the ACS guidelines should be, a frequent statement by consumers (6/17, 35 %) was the belief that guidelines help doctors in their decision making and ensures the provision of the best possible care to every woman and her baby (3/17, 18 %).

*“To provide best practice information/guidance for care providers” (site 3, participant 4)*

*“To provide the best care, informed treatment for both the practitioner and the patient” (site 3, participant 7).*

Consumers felt that a key enabler of CPGs is that they reduce reliance on individual health professionals’ decision making and facilitate consistency of care among health care providers *(*7/17, 41 %*).*

*“To provide a consistency of care” (site 3, participant 1) (In response to the question: What do you think the purpose of the antenatal corticosteroid clinical practice guideline should be)*

Within this domain one barrier identified was the belief by some consumers that health professionals should use guidelines to assist in their decision making but also felt there should be flexibility to allow care to be individualised when necessary *(*3/17, 18 %*).*

*“I think they should use guidelines as well as own experience/knowledge as I understand that all pregnancies are different” (site 2, participant 7).*

## Discussion

In this study the validated TDF helped to assess consumer’s views and attitudes on the administration of ACS and use of a CPG. An extensive literature search, has demonstrated that although the TDF has been used among consumers to assess behaviours related to accessing health services and acceptability of interventions for the prevention or treatment of diseases [39–44], this is the first study to our knowledge to use the TDF to understand consumers’ beliefs towards the receipt of ACS.

Despite a relatively modest sample size, data saturation was reached with the number of participants included in the study. Our study population was representative of the cohort of mothers and babies admitted to neonatal units across New Zealand in relation to social diversity and indications or reasons for preterm birth, when compared with data from the Australia and New Zealand Neonatal Network data (ANZNN 2013), therefore ensuring wide applicability of results. A limitation of our study is that due to the modest sample size we weren’t able to assess differences between different ethnic groups or study locations.

Our study demonstrated that women’s knowledge on why ACS had been administered and the number of courses they had received was very comprehensive. Women demonstrated significant optimism in receipt of ACS and reported belief in the consequences that administration of ACS improved the outcome of their preterm babies.

The main barrier identified by consumers in the receipt of ACS was within the behavioural domain of environmental context and resources. Time constraints and the timely provision of information were perceived by women as key factors hindering the receipt and retention of information on ACS. Consumers’ reported that memory, attention and decision making would be improved by provision of more information on ACS. Furthermore within the domain of behavioural regulation consumers expressed that once they have been identified as at high risk of preterm birth, information should be provided as soon as possible and ideally in both written and verbal formats to facilitate their decision making. This was particularly evident in women who had been identified at risk of preterm birth at very early gestations and were often admitted to hospital for prolonged periods. Consumers believed that availability and provision of this information would help both them and the health professionals who were providing care in an often acute or time pressured situation.

These beliefs were closely linked to the behavioural domain of emotion. Many women expressed that the predominant emotions associated with being identified at risk or being in preterm labour were fear and anxiety. This strongly impacted on their ability to retain any information that was provided. An earlier qualitative study to identify how women cope with the stress of preterm labour found that stress was attributed to feelings of nervousness related to their situation, the lack of control to important aspects in one’s life, and the happening of unexpected events [50]. In attempting to deal with potentially negative situations many individuals struggle to encode and evaluate the information provided into memory and focus instead on bracing themselves for an undesired outcome [51].

A number of women in our study reported that the provision of information or recollection of a discussion on ACS prior to being in preterm labour helped generate confidence in their decision making. These findings are supported by studies which investigated the impact of varying amounts of information prior to diagnostic tests. For example in a qualitative study assessing women’s perspectives of the fetal fibronectin test to assess risk of preterm labour women’s anxiety related to preterm labour was diminished when women received a clear explanation of the potential outcomes prior to the test [52].

Within the domain of social influences, a large proportion of women reported relying on members of the health care team when making decisions about their care and the care of their babies. In particular doctors, nurses and midwives were who consumers reported relying on most. This is consistent with other studies that have identified health care providers as the most important source of information in pregnancy [52–55]. Many consumers felt empowered by these discussions and believed in their own capabilities in using this information to make decisions about their care. This is particularly important as studies have demonstrated that women of childbearing age are very engaged group who often report wanting more information and who like to be actively involved in any decision making [56–59]. Unfortunately, in a limited number of cases, the converse was true and consumers reported feeling disengaged from the decision making and that the decision on receiving ACS was made by their health professionals and not themselves, suggesting a need for improvement. This is critically important in the context of shared decision making as there are numerous afforded benefits to the patient and the health professional when patients feel that they have made an informed choice [60, 61]. These include: improved patient-physician relationships [62]; increased sense of responsibility for themselves and their baby [63]; improved satisfaction [63–66] and lower levels of fear [67].

A number of women reported relying on family and friends for peer support in their decision making. This suggests that to facilitate appropriate ACS administration efforts should be made to include not only women but also their partners and other family members when providing information and discussing administration of ACS. In addition studies in the literature have identified that social support can help patients feel an increased sense of perceived control over a health condition and help manage the uncertainty [68, 69], which would seem particularly important to women at risk of preterm birth.

Through the process of this study, we have elicited consumer’s beliefs and knowledge of a CPG. A large proportion of women who participated in the study had knowledge of the existence of CPGs. However participants often reflected the purpose of a CPG to their use among health care organisations and health professional rather than to themselves. Overall, the majority of women viewed CPGs in a positive light. However a number of consumers believed that there needed to be flexibility to allow their health professionals to individualise care when they felt appropriate. This suggests the need to emphasise to consumers where the recommendations and evidence from CPGs are generated from in the CPG itself or in accompanying patient information materials.

Despite a significant number of participants expressing the need for further information, or clarification related to ACS, no participants commented that they would access a CPG to attempt to address their concerns. These findings are supported by the systematic review conducted to assess patient and public attitudes to and awareness of CPGs [25]. This systematic review reported that several studies had identified that individuals expressed concern that guidelines did not personally help them and were not necessarily applicable to their particular needs. This identifies the ongoing need for guideline groups to engage with consumers and consumer groups in the development of clinical practice guidelines to ensure applicability and to foster confidence in clinical practice guidelines and their recommendations.

Through the process of this study we have identified significant behavioural determinants to be modified or addressed in any guideline implementation strategy. Key functions to an implementation strategy could include training to assist health professionals in providing comprehensive information on ACS and their administration to consumers. Also, providing feedback to health professionals that women identified at risk of preterm birth requested the provision of information as soon as possible, in both written and verbal formats, to facilitate sufficient understanding and retention of the information provided. The use of decision aids [70–72] and interactive health communications [73] have demonstrated positive effects on users particularly in increasing knowledge and reducing decisional uncertainty. However consideration is needed on how and when this information can best be delivered [70, 74]. In the context of our study, women reported being able to recall information on ACS or even hearing of “ACS” prior to being offered them in the acute situation enabled them to feel more confident in their decision making suggest the need to inform health professionals that this information should be provided as soon as possible..

Involving consumers in the development of patient information leaflets would ensure that there is sufficient detail provided to address their uncertainties and concerns and ensure the information is readable and understandable. The results from this study suggest that the implementation strategy needs to include the patient and members of her family to enable social support in stressful situations. Any information should be provided to all individuals involved in the consumers’ decision making processes.

Implementation interventions could focus on educating consumers on the existence of the relevant CPG and their applicability to them and to facilitate use by them and their families. This could be facilitated by the use of posters in clinical areas frequented by consumers explaining how guidelines are synthesised and how they can be accessed and used to facilitate shared decision making.

Further research needs to be undertaken to identify which barriers and enablers to target and to map these barriers and enablers to appropriate intervention functions [75] and behavioural change techniques [76].

## Conclusions

Our study has identified the barriers and enablers to receipt of ACS and use of CPGs, as perceived by New Zealand consumers. The information gathered by our study provides new information on consumer’s views regarding the receipt of ACS including the need to be more actively involved in decision making and for the information provided to be more detailed and delivered in a timely manner. This study adds to the current literature relating to consumers views on the use of CPGs and reiterates the need for consumers to be more actively involved in CPG development and to encourage their use among consumers. The information generated from our study will be used to develop methodologically rigorous intervention strategies and ultimately facilitate implementation of the new ACS clinical practice guidelines.

## Additional file

Additional file 1: Question Guide informed by the Theoretical Domains Framework [36, 38]. (DOCX 16 kb)

## Abbreviations

ACS: Antenatal corticosteroids
CPG: Clinical practice guideline
DHB: District Health Board
TDF: Theoretical domains framework
