# Supplementary material for: Consumers attitudes and beliefs towards the receipt of antenatal corticosteroids and use of clinical practice guidelines
Source: BMC Pregnancy Childbirth. 2016 Sep 5;16(1):259. doi: 10.1186/s12884-016-1043-4 (PMC5011343; doi:10.1186/s12884-016-1043-4)
Supplement: Additional file 1: — Question Guide informed by the Theoretical Domains Framework [36, 38]. (DOCX 16 kb) [file 12884_2016_1043_MOESM1_ESM.docx]

Appendix 1: Questions used to identify consumers attitudes and beliefs towards the receipt of antenatal corticosteroids and the use of clinical practice guidelines and corresponding theoretical domain. ^(36, 38)^

Q1-6 Demographic questions: Age; Ethnicity; Employment; Profession

| Questions for Consumers | Behavioural Domain | |
| --- | --- | --- |
| 1. Are you aware there is a new binational New Zealand and Australian Antenatal Corticosteroid Guideline being produced? | Knowledge | |
| 2. Do you know why antenatal corticosteroids are used in patients at risk of preterm birth? | Knowledge |  |
| 3. In a future pregnancy would you be happy to receive a single course of antenatal corticosteroids again, if you were at risk of your baby being born preterm in the next 24 hours? | Belief about consequences |  |
| 4. In a future pregnancy would you be happy receiving repeated course/s of antenatal corticosteroids if you were at continued risk of your baby being born preterm? | Belief about consequences |  |
| 5. Are there any reasons why you would NOT be happy to receive antenatal corticosteroids? | Belief about consequences |  |
| 6. How many courses of antenatal corticosteroids did you receive? (A complete course is usually 2 injections with betamethasone or 4 injections with dexamethasone). Please comment if you don't know. | Knowledge |  |
| 7. Do you know the name and dose of the antenatal corticosteroids that you were given? | Memory, attention and decision making processes |  |
| 8. Did you receive a complete course (a complete course is 2 injections for betamethasone and 4 injections for dexamethasone) | Memory, attention and decision making processes |  |
| 9. How far on in your pregnancy where you when you were given your first course (injections) of antenatal corticosteroids? | Memory, attention and decision making processes |  |
| 10. How far on in your pregnancy where you when you were given your next course of antenatal corticosteroids? | Memory, attention and decision making processes |  |
| 11. Where you affected by any other medical problems in pregnancy including:   \| High Blood pressure/ Pre-eclampsia \| \| --- \| \| Diabetes \| \| Premature rupture of membranes \| \| Other \| | Knowledge |  |
| 12. Do you think Doctors and Midwives should use clinical practice guidelines when they are making decisions about your care? | Belief about capabilities |  |
| 13. If you had time to consider your treatment options, who would help you in your decision to receive antenatal corticosteroids? | Social influences |  |
| 14. Who would be most important in helping you make decisions about receiving antenatal corticosteroids? | Belief about capabilities |  |
| 15. What do you think the purpose of the antenatal corticosteroid clinical practice guideline should be? | Social professional role and identity |  |
| 15. Were you given any information/explanation prior to receiving antenatal corticosteroids? | Memory, attention and decision processes |  |
| 16. In a future pregnancy would you find it helpful to receive more information? | Behavioural regulation |  |
